# Inhibitory Effect of Interleukin-24 on Programmed Death Ligand 1 Expression via a Eukaryotic Translation Initiation Factor 2 Alpha Kinase 2-Dependent Pathway in Human Triple-Negative Breast Cancer

**DOI:** 10.3390/genes17030339

**Published:** 2026-03-19

**Authors:** Simira Smith, Anastassiya Kim, Alphons Sony, Maryam Aslam, Elouise Torruella, Columba de la Parra, Moira Sauane

**Affiliations:** 1 Department of Biological Sciences, Herbert H. Lehman College, City University of New York, 250 Bedford Park Boulevard West, Bronx, NY 10468, USA; simira.smith@lc.cuny.edu (S.S.); anastassiya.kim@lehman.cuny.edu (A.K.); alphons.sony@lc.cuny.edu (A.S.); maryam.aslam@lehman.cuny.edu (M.A.); elouise.torruella@lc.cuny.edu (E.T.); 2 Ph.D. Program in Biology, The Graduate Center, City University of New York, 365 Fifth Avenue, New York, NY 10016, USA; columba.delaparra@lehman.cuny.edu; 3 Department of Chemistry, Herbert H. Lehman College, City University of New York, 250 Bedford Park Boulevard West, Bronx, NY 10468, USA

**Keywords:** gene therapy, IL-24, breast cancer, triple-negative breast cancer, PD-L1, doxorubicin, EIF2AK2, PKR, apoptosis

## Abstract

Background**/**Objectives: Programmed death ligand 1 (PD-L1) is often overexpressed in triple-negative breast cancer (TNBC), where it helps the tumor evade the immune system and promotes tumor growth. Interleukin-24 (IL-24) is recognized for its anti-tumor activity, although its role in immune regulation remains unclear. In this study, we examined the role of IL-24 in regulating PD-L1 and its anti-cancer activity in TNBC cells. Methods: The study used TNBC cell lines treated with IL-24, delivered via a non-replicating adenovirus vector expressing the *IL-24* gene. Assays included MTT for cell viability, Annexin V for apoptosis, Western blot for protein analysis, and qRT-PCR for mRNA analysis. Results: We found that the highly aggressive MDA-MB-231 cells had significantly higher PD-L1 levels. We discovered that treatment with IL-24 reduced cell growth, induced apoptosis, and significantly decreased PD-L1 protein levels in MDA-MB-231 cells. Mechanistically, we identified PKR, also known as eukaryotic translation initiation factor 2 alpha kinase 2, as a key mediator of IL-24–induced PD-L1 suppression. Additionally, doxorubicin, a primary chemotherapy drug used to treat triple-negative breast cancer, decreases PD-L1 expression and increases the sensitivity when combined with IL-24. Conclusions: In this study, we show that IL-24 decreases PD-L1 expression in MDA-MB-231 cells through PKR activation, enhances the anti-tumor effects of Doxorubicin, and may enable lower doses that reduce toxicity and further decrease PD-L1 levels. These findings suggest that IL-24 could serve as a valuable target for therapeutic intervention and suggest that it can improve doxorubicin’s effectiveness against aggressive breast cancer.

## 1. Introduction

Immune checkpoint pathways are crucial regulators of antitumor immunity, and blocking the PD-1 (Programmed Cell Death Protein 1)/PD-L1 (Programmed Death-Ligand 1) axis has transformed treatment for many cancers. However, many patients either do not respond to or develop resistance to PD-1/PD-L1 inhibitors, emphasizing the need to understand how PD-L1 expression is regulated in tumor cells and to develop strategies to modulate these pathways for improved immunotherapy effectiveness [1]. Interleukin-24 (IL-24) is a tumor-suppressing cytokine that triggers apoptosis in a wide variety of cancer cells through multiple, mostly cancer-specific signaling pathways [2]. In various types of tumors, IL-24 stimulates stress-activated protein kinases, most notably p38 mitogen-activated protein kinase and c-Jun N-terminal kinase, through signaling pathways that operate independently of the classical Janus kinase/signal transducer and activator of transcription (JAK/STAT) pathway. This alternative mechanism upregulates the expression of growth arrest and DNA damage-inducible (GADD) genes, and activates executioner caspases, thereby selectively inducing cancer-specific apoptosis [3,4,5]. IL-24 triggers endoplasmic reticulum (ER) stress, resulting in the upregulation of key ER stress markers such as BiP/GRP78, GRP94, XBP1, and eIF2α. IL-24 also triggers mitochondrial impairment, marked by a reduction in membrane potential, activation of Bax, release of cytochrome c, elevated reactive oxygen species (ROS) levels, and activation of caspase-3, ultimately triggering the intrinsic pathway of apoptosis [6]. In various cancers, IL-24-driven activation of p38 MAPK boosts ROS production while suppressing Nrf2-mediated antioxidant defenses, intensifying oxidative damage [7]. Simultaneously, IL-24 activates protein kinase A (PKA), which phosphorylates and inhibits glycogen synthase kinase 3β (GSK3β), disrupting glucose metabolism and exacerbating metabolic stress, ultimately causing cell death [8]. Both extracellular and intracellular IL-24 function through JAK/STAT-independent pathways that depend on p38 MAPK, connecting ER stress to mitochondrial dysfunction. IL-24 induces tumor-specific cytotoxicity while sparing normal cells; these findings suggest that IL-24 could serve as a valuable target for therapeutic intervention that exploits apoptosis-related vulnerabilities in cancer [9]. Autophagy and apoptosis are two critical stress-adaptive processes that influence tumor cell fate and response to therapy [10]. In many cancers, autophagy helps cells survive during metabolic or treatment-induced stress, while apoptosis is often suppressed, leading to treatment resistance [10]. IL-24 induces ER stress and ceramide production, which initiate early-stage autophagy as a protective response. As ER stress and ROS levels increase, IL-24 signaling shifts from promoting autophagy to inducing apoptosis [11]. PKR has been linked to both apoptotic and autophagic pathways, and IL-24 has been shown to enhance cancer cell death [12]. However, how IL-24–PKR signaling coordinates these processes in immune checkpoint control remains unclear.

This study investigates the relationship between IL-24, PKR signaling, and PD-L1 regulation in triple-negative breast cancer (TNBC). The findings show, for the first time, that IL-24 decreases PD-L1 mRNA and protein levels, and this reduction may be mediated through the PKR pathway. These results establish, for the first time, an IL-24–PKR–PD-L1 signaling pathway that links immune checkpoint regulation. Additionally, our findings demonstrate that IL-24 enhances the antitumor effects of doxorubicin and may enable lower doses, thereby reducing toxicity while decreasing PD-L1 expression in MDA-MB-231 cells. In summary, IL-24 promotes tumor suppression and lowers PD-L1 expression. Moreover, our results suggest that combining low-dose IL-24 with doxorubicin enhances antitumor responses while reducing PD-L1 expression, offering new strategies for immune checkpoint blockade.

## 2. Materials and Methods

**Cells and Culture Conditions.** All the breast cancer cell lines in this study, T47D (ATCC HTB-133), MCF7 (ATCC HTB-22), MDA-MB-453 (ATCC HTB-131), and MDA-MB-231 (ATCC HTB-26) are standard human breast cancer cell lines commonly used in cancer research, each representing distinct breast cancer subtypes and exhibiting varying characteristics, including hormone receptor status, epithelial or mesenchymal phenotype, and invasiveness. MCF7 and T47D are ER-positive breast cancer cell lines, MDA-MB-231 is a triple-negative line with a mesenchymal phenotype, and MDA-MB-453 is HER2-positive. All cell lines were obtained from the ATCC (Manassas, VA, USA) and cultured according to ATCC recommendations. Experiments used cultures between passages 3 and 16, counted from the initial thaw; cultures beyond passage 16 were not utilized. Cell line identity was verified through short tandem repeat (STR) analysis using a human STR marker panel, and the profiles were compared with ATCC records to confirm correct identity and avoid cross-contamination. Cell cultures were routinely screened for Mycoplasma contamination using a commercially available PCR-based detection kit (ab289834, Abcam, Cambridge, MA, USA); only mycoplasma-negative cells were used for experiments. All cell lines were incubated in a humidified at 37 °C with 5% CO_2_ atmosphere, with medium changed every other day. C16 (PKR inhibitor; Tocris Bioscience (Bristol, UK) cat. no. 5382; CAS 608512-97-6; ≥98% purity by HPLC; MW 268.29) was purchased from Tocris Bioscience. C16 powder was dissolved in cell culture-grade DMSO (Sigma-Aldrich D4540, Waltham, MA, USA) to yield a 50 mM stock solution, per the manufacturer’s specified maximum solubility in DMSO. Stocks were aliquoted, stored at −20 °C, and used within their stated stability window. For experiments, a working dilution (0.5 μM) was prepared fresh in full growth medium from stock, ensuring final DMSO levels stayed at or below 0.1% (*v*/*v*); matching vehicle controls included equivalent DMSO volumes.

**Adenovirus transduction.** The viruses were purchased from VectorBuilder, Inc. (Chicago, IL, USA). Cells were plated at 20% confluence in suitable culture vessels 24 h before transduction. Replication-deficient recombinant adenoviruses (VectorBuilder, Inc., Chicago, IL, USA), either the IL-24-expressing, replication-defective adenovirus (Ad.IL-24) or a control, an identical empty adenovirus vector lacking foreign genes (Ad.vector), were diluted in serum-free medium following the manufacturer’s protocol, determined empirically to optimize efficiency and minimize toxicity. Transduction proceeded for 1–2 h at 37 °C, followed by medium replacement with 10% serum-containing growth medium, as described previously [8,13]. To optimize transduction efficiency while minimizing cytotoxicity, we tested multiplicities of infection (MOI) in MDA-MB-231 cells (ATCC HTB-26). Based on these evaluations, an MOI of 180 was selected for all subsequent experiments. In contrast, infection at 75 MOI did not cause detectable apoptosis on its own but, when combined with doxorubicin treatment, induced significant apoptosis. MDA-MB-231 cells were seeded at 2.5 × 10^5^ cells per well in 6-well plates containing 2 mL of serum-free DMEM and allowed to adhere overnight. Cells were then treated with a non-replicative adenovirus vector (titer: 9.64 × 10^10^ IFU/mL, according to the manufacturer’s certificate of analysis) by adding 3 μL of stock per well, resulting in a final virus concentration of 4.5 × 10^4^ IFU/mL (total IFU = 9 × 10^7^; MOI = 180 based on the seeded cell number). After 2 h of incubation at 37 °C, the medium was replaced with complete growth medium (DMEM supplemented with 10% FBS), and the cells were maintained for 72 h post-infection before analysis.

**Quantitative real-time PCR (qRT-PCR).** Total RNA was isolated from MDA-MB-231 cells using Qiazol lysis reagent (Qiagen, Venlo, The Netherlands). RNA quantity and purity were assessed using a NanoDrop 2000 spectrophotometer (Thermo Fisher Scientific, Saint Louis, MO, USA). cDNA was synthesized from 1 μg total RNA using Applied Biosystems™ High-Capacity cDNA Reverse Transcription Kit, per manufacturer’s protocol. Quantitative PCR was carried out using SYBR Green master mix from GlpBio on a BioRad CFX-connect Real-time PCR thermocycler (Hercules, CA, USA). Each reaction contained 20 ng of cDNA in 20 μL of total reaction volume. The thermocycling conditions included initial denaturation at 95.0 °C, followed by 40 cycles of denaturation, annealing, and extension. Target gene expression was normalized to GAPDH, with relative quantification determined by the 2^−ΔΔCt^ method. Reactions were run in duplicate with 3 biological replicates using qPCR primers from IDT (Coralville, IA, USA).

**Protein extraction and immunoblot analysis.** Cell lysates were prepared in radio-immunoprecipitation assay (RIPA) buffer containing a broad-spectrum protease inhibitor cocktail. Protein concentration was measured, and 50 µg of total protein per sample was separated by SDS–PAGE on 12% polyacrylamide gels, then transferred onto nitrocellulose membranes. Membranes were blocked with Odyssey Blocking Buffer (LI-COR Biosciences) and incubated overnight at 4 °C with the indicated primary monoclonal antibodies. The following antibodies were used: anti–PD-L1 (Proteintech, 66248-1-Ig, 1:1000, 40–50 kDa), anti–phospho-PKR (Thr446) (Sigma-Aldrich, catalog number 07-532, dilution 1:1000, 70 kDa), anti–IL-24 (R&D Systems (Minneapolis, MN, USA), catalog number AF1965-SP, dilution 1:1000, ~18–40 kDa), and anti–β-actin (Cell Signaling Technology (Danvers, MA, USA), catalog number 3700, dilution 1:2000, 42 kDa). After washing, membranes were probed for 45 min at room temperature with infrared dye-conjugated secondary antibodies—goat anti-rabbit IgG (H+L) 800 CW, goat anti-rabbit 680 RD, or goat anti-mouse IgG (H + L)—each diluted 1:25,000 in Odyssey blocking buffer. Membranes were then washed with PBS-0.1% Tween-20. Bands were imaged and quantified using the Odyssey CLx Imaging System and Image Studio software (version 5.2, LI-COR Biosciences, Lincoln, NE, USA). PD-L1 expression was normalized to β-actin, and data are presented as mean ± S.D. from at least three independent experiments.

**Viability Assay.** Cells were seeded with DMEM supplemented with 10% FBS and allowed to adhere at a density of 2 × 10^3^ cells per well in 96-well plates for 12 h prior to the treatment. Cell viability was determined using the MTT 3-(4,5-dimethylthiazol-2-yl)-2,5-diphenyltetrazolium bromide assay following standard procedures. After incubation with MTT reagent, formazan crystals were solubilized, and absorbance was recorded at 595 nm using a microplate reader. The optical density values were proportional to the number of metabolically active (viable) cells.

**Apoptosis Assay.** Cells were detached with Accutase, washed once with complete medium and PBS containing 10% FBS, then resuspended in 96 μL Annexin V binding buffer. Samples were stained with 1.5 μL of Annexin V-FITC and 12 μL of propidium iodide (PI) for 15 min on ice (Cell Signaling Technology, Inc., Danvers, MA, USA). Approximately 600,000 cells were stained per sample. However, flow cytometry was running until 35,000 cells in the Single cell gate were analyzed. Analysis was done using BD Accuri C6 Plus cell analyzer (Dickinson and Co., Franklin Lakes, NJ, USA).

**Statistical Analysis.** Results are reported as mean ± standard error of the mean (SEM) from a minimum of three independent biological experiments. Data analyses were performed with GraphPad Prism 10 (GraphPad Software, San Diego, CA, USA) and Microsoft Excel. Pairwise comparisons used unpaired, two-tailed Student’s *t*-text, while multi-group analyses applied two-way ANOVA, followed by Tukey’s post hoc test. Differences were considered significant at *p* < 0.05 (* *p* < 0.05, ** *p* < 0.01, *** *p* < 0.001, **** *p* < 0.0001, n.s., not significant) and 95% confidence intervals were calculated for primary endpoints, with asterisks marking differences form controls.

## 3. Results

### 3.1. Analysis of PD-L1 Expression in Triple-Negative (MDA-MB-231), ER-Positive (MCF-7, T47D), and HER2-Positive (MDA-MB-453) Cell Lines

**Figure 1:**
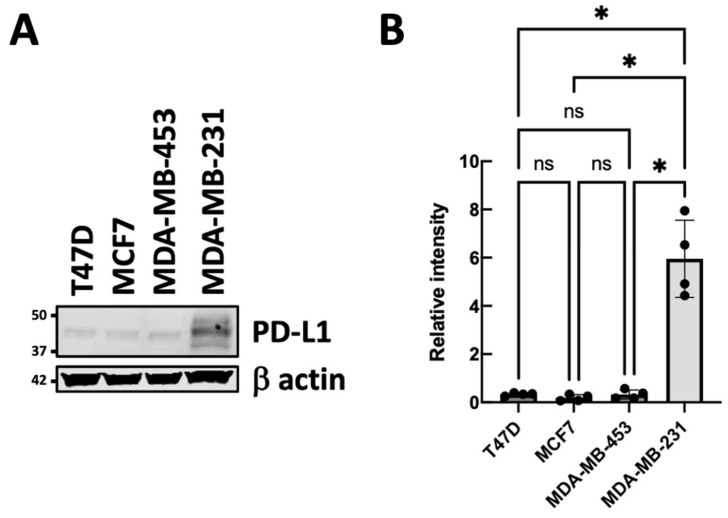
Comparison of PD-L1 protein levels across breast cancer cell lines. Western blot and quantification analyses of PD-L1 levels were performed on T47D, MCF7, MDA-MB-231, and MDA-MB-453 cell lines. (**A**) Cells were collected, proteins purified, and Western blot analysis was conducted to measure PD-L1 levels in these cell lines. β-actin was used as a loading control. The figure shown is representative of four independent experiments. (**B**) Representative Western blot showing PD-L1 protein levels under the indicated experimental conditions. Band intensities were quantified using the Odyssey Imaging System software v.5.2 (LI-COR Biosciences, Lincoln, NE, USA), and PD-L1 expression in each sample was normalized to the corresponding β-actin signal. The bar graph shows the mean ± SEM from four independent experiments. Group comparisons were conducted with one-way ANOVA and subsequent Tukey’s post hoc test. * *p* < 0.05, ns: not significant.

We first evaluated PD-L1 expression across multiple breast cancer cell lines. As shown in Figure 1, Western blot analysis detected PD-L1 protein in the triple-negative breast cancer line MDA-MB-231. The highly aggressive MDA-MB-231 cells showed significantly higher PD-L1 protein expression compared to other breast cancer cell lines (T47D, MCF7, and MDA-MB-453).

### 3.2. IL-24 Decreases Both PD-L1 mRNA and Protein Levels

**Figure 2:**
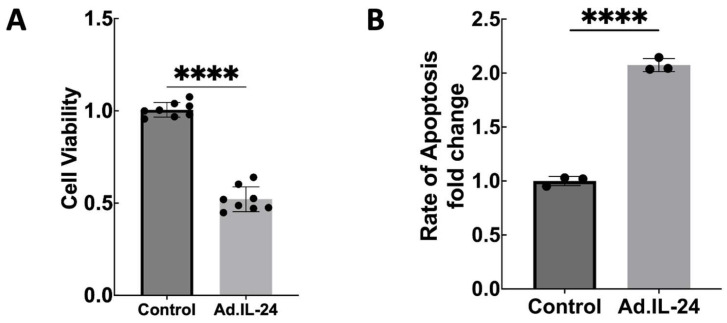
IL-24 regulates proliferation and apoptosis in MDA-MB-231 cells. (**A**) MDA MB 231 cells were treated with non-replicative virus (MOI 180) encoding IL-24–expressing or control (Ad.vector) constructs, and cell viability was evaluated 5 days post-treatment using the MTT assay. Data are expressed as the ratio of treated to control cells. Statistical significance was determined by Welch’s *t*-test. The figure shown is representative of three independent experiments. Values represent the mean ± S.D., calculated from combined data from technical samples across independent experiments. (**B**) Cells treated as in (**A**) were evaluated for apoptosis via Annexin V staining and analyzed by flow cytometry (FACS) 72 h after infection using v.64-bit BD Accuris C6 Plus software (Becton Dickinson, Berkshire, UK). Data show the average apoptosis rate, normalized to the specified control (±SD) across three separate biological replicates. Statistical significance was assessed using an unpaired two-tailed Student’s *t*-test. **** *p* < 0.0001.

**Figure 3:**
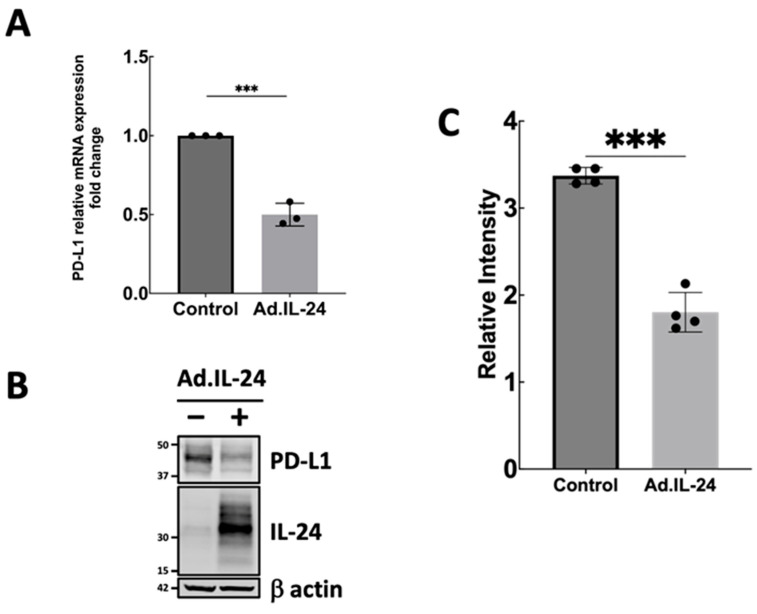
Effect of IL-24 on PD-L1 expression in MDA-MB-231 cells. (**A**) MDA-MB-231 cells were treated with non-replicative virus (MOI 180) encoding IL-24–expressing or control (Ad.vector) constructs for 72 h, and mRNA levels of PD-L1 expression were analyzed using qRT-PCR. Data represent mean fold change in relative expression change calculated using the 2^−ΔΔCt^ method (±SD) from three independent biological replicates. GAPDH was used as a housekeeping gene for normalization. Statistical significance between groups was assessed using an unpaired two-tailed Student’s *t*-test on Δ*Ct* values. (**B**) PD-L1 protein levels were analyzed by Western blot after treatment with or without IL-24 as described in A. The figure shown is representative of three independent experiments. (**C**) Quantification of PD-L1 protein levels normalized to β-actin from Western blot. Statistical significance was determined by Welch’s *t*-test. The figure shown is representative of four independent experiments. *** *p* < 0.001.

First, cell viability and induction of apoptosis in MDA-MB-231 triple-negative breast cancer cells exposed to IL-24 were quantified by MTT assay and Annexin V–FITC/propidium iodide flow cytometry, respectively. Cytotoxic responses to IL-24 treatment are depicted in Figure 2 and Supplementary Figures S1–S3. The cytotoxic effect of IL-24 on the MDA-MB-231 human breast cancer cell line is shown in Figure 2 and Figure S1–S3. To determine whether IL-24 affects PD-L1 expression in breast cancer cells, MDA-MB-231 cells were treated with IL-24 (Ad.IL-24) or control (Ad.vector). PD-L1 mRNA and protein levels were measured by quantitative real-time PCR and Western blotting, respectively. As shown in Figure 3, IL-24 treatment significantly reduced PD-L1 mRNA (Figure 3A and Figure S4) and protein levels compared to the control (Figure 3B,C). These findings suggest that IL-24 suppresses PD-L1 expression at both the mRNA and protein levels in the aggressive MDA-MB-231 breast cancer cell line.

### 3.3. IL-24 Reduces PD-L1 Protein Levels by Activating the Protein Kinase R (PKR) Pathway

**Figure 4:**
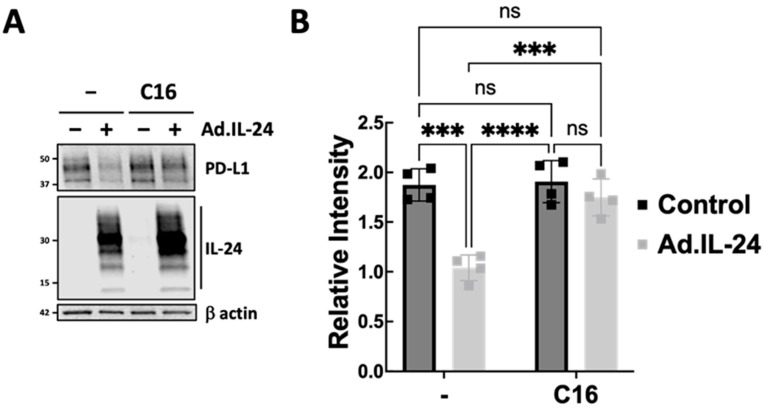
IL-24 reduces PD-L1 expression by activating PKR**.** MDA-MB-231 cancer cells were treated with either a non-replicative virus (MOI 180) encoding IL-24–expressing constructs or a control (Ad.vector) construct, C16 (a PKR inhibitor), or both. (**A**) PD-L1 protein levels were measured by Western blot after treatment with or without IL-24 and C16. The figure shown is representative of four independent experiments. (**B**) Western blot quantification of PD-L1 relative to β-actin was performed using Odyssey Imaging System software v.5.2. Comparisons between groups were done with two-way ANOVA, followed by Tukey’s post hoc test. *** *p* < 0.001, **** *p* < 0.0001, ns: not significant.

Although PD-1/PD-L1 inhibitors have shown effectiveness in treating triple-negative breast cancer (TNBC), resistance—both primary and acquired—often complicates outcomes [14]. This resistance usually arises from abnormal PD-L1 expression on tumor cells, highlighting the importance of identifying key molecular regulators of this pathway. Prior research indicates that interleukin-24 (IL-24) activates PKR in various cancer cell lines, including breast cancer models [4,15,16]. We and others have shown that IL-24 causes phosphorylation of eukaryotic initiation factor 2α (eIF2α), a main downstream target of PKR. This triggers an integrated stress response (ISR) that promotes tumor cell death while protecting normal tissues [16,17,18]. It is well established that PKR activation leads to eIF2α phosphorylation at serine 51, which inhibits overall protein synthesis but increases the expression of specific ISR effectors, such as ATF4 and CHOP [19,20]. These effectors influence signaling pathways, including those that destabilize PD-L1 mRNA and reduce protein levels [16,21]. Given that PD-L1 overexpression supports immune evasion and resistance to therapy in TNBC [22,23], we investigated whether IL-24 could reduce PD-L1 through the PKR pathway, opening potential for new immunotherapy approaches. The compound C16, a specific ATP-competitive inhibitor of PKR, effectively blocks its autophosphorylation. As shown in Figure 4, C16 significantly prevented IL-24-induced decreases in PD-L1 levels in the MDA-MB-231 breast cancer cell line. Treatment with IL-24 induces PKR phosphorylation, but this effect is blocked by pretreatment with C16 (Figure S5). These results suggest that PKR activation may be essential for IL-24 to lower PD-L1 levels.

### 3.4. The Combination of Low Concentrations of Interleukin-24 (IL-24) with Doxorubicin (DOX) Significantly Decreases PD-L1 Expression and Enhances Chemosensitivity in MDA-MB-231 Triple-Negative Breast Cancer (TNBC) Cells

**Figure 5:**
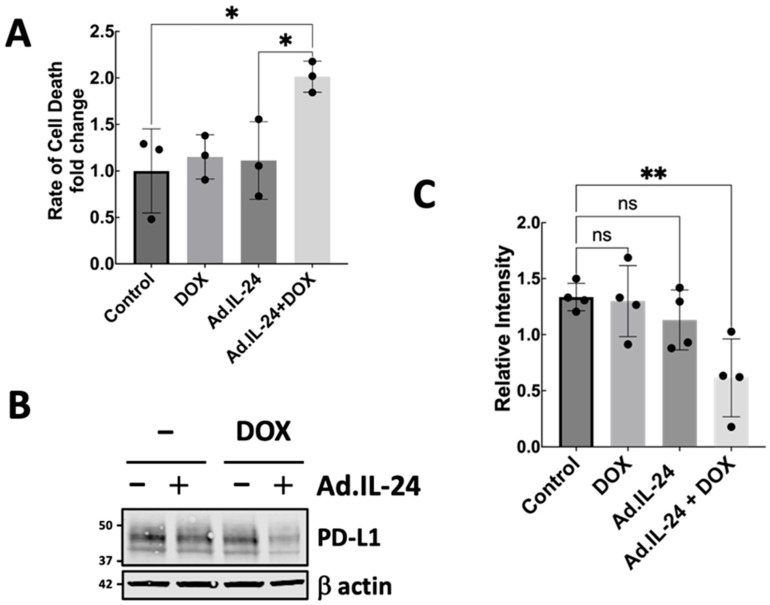
Combined effects of doxorubicin (DOX) and IL-24 on PD-L1 expression and apoptosis in MDA-MB-231 triple-negative breast cancer cells. (**A**) MDA-MB-231 cancer cells were treated for 72 h with low doses of DOX (0.1 μM) or low doses of a non-replicative virus (MOI 75) encoding an IL-24–expressing construct, or both. Cells were evaluated for apoptosis via Annexin V staining and analyzed by flow cytometry (FACS) 72 h after infection, using CellQuest software (Becton Dickinson). Data represent the mean rate of cell death normalized to the indicated control (±SD) from three independent biological replicates. Statistical analysis was performed using unpaired ordinary one-way ANOVA. (**B**) PD-L1 protein levels were measured by Western blot after treatment with or without IL-24 and DOX. The figure shown is representative of four independent experiments. (**C**) Western blot quantification of PD-L1 relative to β-actin was performed using Odyssey Imaging System software. Statistical comparisons between groups were performed using two-way ANOVA followed by Tukey’s post hoc test. * *p* < 0.05, ** *p* < 0.01, ns: not significant.

Doxorubicin (DOX), a commonly used chemotherapy drug for TNBC, effectively slows tumor growth. However, its clinical application is limited by cumulative, dose-dependent cardiotoxicity and subsequent cardiomyopathy, as well as other systemic side effects [24]. IL-24 induces apoptosis in various cancers, including breast cancer cells, both alone and when combined with other therapies [4,9,18,21]. The combination of low-dose IL-24 (MOI 75) with low-dose DOX (0.1 μM) produces a stronger apoptotic and cytotoxic response than either treatment alone (Figure 5A). Additionally, this combination more effectively reduces PD-L1 expression than either agent alone (Figure 5B). These results suggest that IL-24 enhances the anti-tumor effects of DOX and could permit lower doses, potentially reducing toxicity while also decreasing PD-L1 expression in TNBC.

## 4. Discussion

Triple-negative breast cancer (TNBC) is one of the most resistant breast cancer subtypes to treatment, exhibiting high recurrence rates and poor clinical outcomes despite the use of surgery, radiotherapy, chemotherapy, and emerging targeted therapies [25,26,27]. The lack of estrogen receptor (ER), progesterone receptor (PR), and human epidermal growth factor receptor 2 (HER2) expression prevents the use of hormone and HER2-targeted treatments, leaving most patients dependent on cytotoxic therapies. Intratumoral heterogeneity, widespread genetic and signaling disruptions, increased drug efflux activity, growth of cancer stem-like cells, and immunosuppressive tumor microenvironments all contribute to resistance to both chemotherapeutic and immunotherapeutic drugs [28,29]. These complex mechanisms drive the aggressive clinical progression and poor prognosis typical of triple-negative breast cancer (TNBC) [30]. Understanding the main signaling pathways and their interactions is crucial for developing biomarker-driven combination therapies [31]. Such targeted approaches have significant potential to overcome resistance and improve treatment outcomes in TNBC [31].

Interleukin 24, a member of the Interleukin-10 family, inhibits tumor growth by inducing apoptosis in various cancers without harming normal cells [4,9,32]. This is supported by preclinical and clinical studies showing selective tumor cell death, objective responses, and a favorable safety profile. This cancer-specific activity has been observed in preclinical models of glioblastoma, colorectal cancer, breast cancer, and other tumors [33]. IL-24 signals through both the IL-20R1/IL-20R2 and IL-22R/IL-20R2 receptor complexes, as well as through receptor-independent intracellular pathways, which appear especially important for cancer cell-specific toxicity. This dual mechanism supports its combined cytokine-like immunomodulatory functions and its role in stress responses and apoptosis within tumor cells [4,9,32]. Future research will determine whether IL-24 downregulates PD-L1 through traditional IL-20 receptor signaling or receptor-independent mechanisms. Mechanistically, IL-24 triggers ER stress and activates the unfolded protein response in cancer cells, including PERK activation, eIF2α phosphorylation, and ATF4/CHOP induction, all closely linked to apoptosis [4]. Additionally, IL-24 stimulates PKR, another eIF2α kinase, in lung and breast tumor cells, positioning PKR as a key component of the stress signaling pathway triggered by IL-24 [18]. IL-24 activates PKR primarily in the cytosol, where it binds to and activates PKR, initiating a PKR-driven inflammatory response under proteotoxic stress. A crucial future step will be to distinguish PKR-dependent from PKR-independent effects of IL-24 across different tumor types, to identify combination strategies that specifically enhance PKR-mediated killing while minimizing toxicity in normal tissues [18,33]. In this study, we identified IL-24 as a regulator of tumor immune evasion through a PKR-dependent mechanism in human TNBC cells. Specifically, IL-24 decreases PD-L1 expression in cancer cells, and this process requires activation of PKR, establishing an IL-24–PKR–PD-L1 axis that links stress signaling to immune checkpoint regulation. To our knowledge, this is the first evidence that IL-24 suppresses PD-L1, and that this suppression is mediated by PKR-dependent mechanisms, suggesting that IL-24 may enhance antitumor immunity by decreasing PD-L1–mediated immune escape. The interaction among IL-24 signaling, PKR activation, and PD-L1 regulation highlights IL-24’s complex role in tumor suppression, combining direct killing of cancer cells with modulation of the immune system. Building on our findings that IL-24 treatment suppresses PD-L1 at both the transcript and protein levels, future experiments will investigate whether IL-24-driven reductions in PD-L1 mRNA and protein also affect cell-surface expression, thereby highlighting its potential to interfere with tumor immune escape. Future in vivo studies and translational research on patient samples will be essential to further explore the IL-24–PKR–PD-L1 pathway, and to identify its upstream and downstream components, in order to improve the effectiveness of treatments for aggressive breast cancers.

Previous research showed that IL-24 acts synergistically with doxorubicin in multidrug-resistant colorectal cancer cells by reversing P-glycoprotein–mediated drug resistance and enhancing doxorubicin-induced cytotoxicity [34]. In this study, we also demonstrate that doxorubicin in triple-negative breast cancer, a subtype with limited treatment options and high recurrence rates, significantly reduces PD-L1 expression and increases chemosensitivity in MDA-MB-231 cells when combined with interleukin-24. Since doxorubicin’s effectiveness is often limited by dose-related toxicities, IL-24’s ability to boost doxorubicin activity without adding to cytotoxic effects offers promising translational potential in treating triple-negative breast cancer. Overall, these results encourage further research into IL-24 and doxorubicin combination therapy as a strategic way to enhance antitumor effects while reducing systemic toxicity in aggressive breast cancers.

## Data Availability

The data are included in the article or in the Supplementary Materials. Data from this study are available upon request to the corresponding author.

## Supplementary Materials

The following supporting information can be downloaded at: https://www.mdpi.com/article/10.3390/genes17030339/s1. Figure S1: Gating strategy and representative apoptotic profiles of MDA-MB-231 cells. Flow cytometric analysis was used to evaluate apoptosis in MDA-MB-231 cells treated with Ad.IL-24 or control vector. Shown are gating steps to exclude debris and doublets, and representative Annexin V/PI plots highlighting increased apoptotic populations in Ad.IL-24-treated cells compared with control. Figure S2: Quantification of apoptosis in Ad.IL-24-treated MDA-MB-231 cells. Flow cytometric analysis based on Annexin V/PI staining shows that Ad.IL-24-treated cells exhibit a larger Annexin V–positive population than controls, indicating a marked increase in both early- and late-apoptotic cells, consistent with the results shown in Figure 2B. Figure S3: Raw data and normalization were used to quantify apoptosis. The table summarizes raw flow cytometry data from three biological replicates showing the distribution of cell populations across the four quadrants of the Annexin V/PI plots. The total apoptotic population was determined by combining early and late apoptotic cells and was used for the analysis in Figure 2B. Values were normalized to the control samples’ mean and reported as fold changes compared to the control. Figure S4: Primer sequences used for qPCR analysis. The table lists the primer sequences for PD-L1 and the housekeeping gene GAPDH obtained from Integrated DNA Technologies (IDT), which were used for quantitative PCR validation of gene expression. Figure S5: Effect of C16 inhibitor on PKR phosphorylation. Western blot analysis shows phosphorylated PKR (p-PKR) and β-actin in MDA-MB-231 cells treated with Ad.IL-24, with or without C16 pretreatment. The results indicate that C16 effectively prevents PKR phosphorylation, as p-PKR levels remain low both after C16 pretreatment alone and following Ad.IL-24 treatment in the presence of C16.

## Abbreviations

The following abbreviations are used in this manuscript:

PD-L1	Programmed Death Ligand 1
TNBC	Triple-negative Breast Cancer
IL-24	Interleukin-24
Ad.IL-24	Non-replicating adenovirus vector expressing the IL-24 gene
Ad.vector	Non-replicating adenovirus vector
PKR	protein kinase R
EIF2AK2	Eukaryotic Translation Initiation Factor 2 alpha kinase 2
PD-1	Programmed Cell Death Protein 1
MAPK	Mitogen-activated Protein Kinase
JNK	c-Jun N-terminal Kinase
JAK	Janus Kinase
STAT	Signal Transducer and Activator of Transcription
GADD	Growth Arrest and DNA Damage-inducible
ER	Endoplasmic Reticulum
ROS	Reactive Oxygen Species
BiP	Binding immunoglobulin Protein
GRP78	Glucose-Regulated Protein 78
GRP94	Glucose-Regulated Protein 94
XBP1	X-box Binding Protein 1
eIF2α	Eukaryotic Translation Initiation Factor 2 Alpha Subunit
Nrf2	Nuclear Factor Erythroid 2-related Factor 2
GSK3β	Glycogen Synthase Kinase 3β
MTT	3-(4,5-dimethylthiazol-2-yl)-2,5-diphenyltetrazolium bromide
FITC	Annexin V-fluorescein Isothiocyanate
PI	Propidium Iodide
ISR	Integrated Stress Response
C16	Compound C16
DOX	Doxorubicin
ATF	Activating Transcription Factor 4

## References

[1] Zhou Y.-J., Li G., Wang J., Liu M., Wang Z., Song Y., Zhang X., Wang X. (2023). PD-L1: Expression regulation. Blood Sci..

[2] Sauane M., Gopalkrishnan R.V., Sarkar D., Su Z.-Z., Lebedeva I.V., Dent P., Pestka S., Fisher P.B. (2003). MDA-7/IL-24: Novel cancer growth suppressing and apoptosis inducing cytokine. Cytokine Growth Factor. Rev..

[3] Sarkar D., Su Z.-Z., Lebedeva I.V., Sauane M., Gopalkrishnan R.V., Valerie K., Dent P., Fisher P.B. (2002). mda-7 (IL-24) Mediates selective apoptosis in human melanoma cells by inducing the coordinated overexpression of the GADD family of genes by means of p38 MAPK. Proc. Natl. Acad. Sci. USA.

[4] Smith S., Lopez S., Kim A., Kasteri J., Olumuyide E., Punu K., de la Parra C., Sauane M. (2023). Interleukin 24: Signal Transduction Pathways. Cancers.

[5] Sauane M., Su Z.-Z., Gupta P., Lebedeva I.V., Dent P., Sarkar D., Fisher P.B. (2008). Autocrine regulation of mda-7/IL-24 mediates cancer-specific apoptosis. Proc. Natl. Acad. Sci. USA.

[6] Gupta P., Su Z.-Z., Lebedeva I.V., Sarkar D., Sauane M., Emdad L., A Bachelor M., Grant S., Curiel D.T., Dent P. (2006). mda-7/IL-24: Multifunctional cancer-specific apoptosis-inducing cytokine. Pharmacol. Ther..

[7] Tian H., Zhang D., Gao Z., Li H., Zhang B., Zhang Q., Li L., Cheng Q., Pei D., Zheng J. (2014). MDA-7/IL-24 inhibits Nrf2-mediated antioxidant response through activation of p38 pathway and inhibition of ERK pathway involved in cancer cell apoptosis. Cancer Gene Ther..

[8] Kim A., Lopez S., Smith S., Sony A., Abreu J., de la Parra C., Sauane M. (2025). Interleukin 24 Promotes Mitochondrial Dysfunction, Glucose Regulation, and Apoptosis by Inactivating Glycogen Synthase Kinase 3 Beta in Human Prostate Cancer Cells. Cells.

[9] Raguraman R., Munshi A., Ramesh R. (2025). Interleukin-24: A Multidimensional Therapeutic for Treatment of Human Diseases. Wiley Interdiscip. Rev. Nanomed. Nanobiotechnol..

[10] Buzun K., Gornowicz A., Lesyk R., Bielawski K., Bielawska A. (2021). Autophagy Modulators in Cancer Therapy. Int. J. Mol. Sci..

[11] Dent P., Yacoub A., Hamed H.A., Park M.A., Dash R., Bhutia S.K., Sarkar D., Gupta P., Emdad L., Lebedeva I.V. (2010). MDA-7/IL-24 as a cancer therapeutic: From bench to bedside. Anticancer Drugs.

[12] Kang R., Tang D. (2012). PKR-dependent inflammatory signals. Sci. Signal..

[13] Persaud L., Mighty J., Zhong X., Francis A., Mendez M., Muharam H., Redenti S.M., Das D., Aktas B.H., Sauane M. (2018). IL-24 Promotes Apoptosis through cAMP-Dependent PKA Pathways in Human Breast Cancer Cells. Int. J. Mol. Sci..

[14] Chen X., Feng L., Huang Y., Wu Y., Xie N. (2022). Mechanisms and Strategies to Overcome PD-1/PD-L1 Blockade Resistance in Triple-Negative Breast Cancer. Cancers.

[15] Zheng M., Bocangel D., Doneske B., Mhashilkar A., Ramesh R., Hunt K.K., Ekmekcioglu S., Sutton R.B., Poindexter N., Grimm E.A. (2007). Human interleukin 24 (MDA-7/IL-24) protein kills breast cancer cells via the IL-20 receptor and is antagonized by IL-10. Cancer Immunol. Immunother..

[16] Davidson S., Yu C.-H., Steiner A., Ebstein F., Baker P.J., Jarur-Chamy V., Schaale K.H., Laohamonthonkul P., Kong K., Calleja D.J. (2022). Protein kinase R is an innate immune sensor of proteotoxic stress via accumulation of cytoplasmic IL-24. Sci. Immunol..

[17] Persaud L., Zhong X., Alvarado G., Do W., Dejoie J., Zybtseva A., Aktas B.H., Sauane M. (2017). eIF2α Phosphorylation Mediates IL24-Induced Apoptosis through Inhibition of Translation. Mol. Cancer Res..

[18] Hu C.-W., Yin G.-F., Wang X.-R., Ren B.-W., Zhang W.-G., Bai Q.-L., Lv Y.-M., Li W.-L., Zhao W.-Q. (2014). IL-24 Induces Apoptosis via Upregulation of RNA-Activated Protein Kinase and Enhances Temozolomide-Induced Apoptosis in Glioma Cells. Oncol. Res..

[19] Lee Y.S., Kunkeaw N., Lee Y.-S. (2020). Protein kinase R and its cellular regulators in cancer: An active player or a surveillant?. Wiley Interdiscip. Rev. RNA.

[20] Tian X., Zhang S., Zhou L., Seyhan A.A., Borrero L.H., Zhang Y., El-Deiry W.S. (2021). Targeting the Integrated Stress Response in Cancer Therapy. Front. Pharmacol..

[21] Li Y.-J., Liu G., Li Y., Vecchiarelli-Federico L.M., Liu J.C., Zacksenhaus E., Shan S.W., Yang B.B., Li Q., Dash R. (2013). mda-7/IL-24 expression inhibits breast cancer through upregulation of growth arrest-specific gene 3 (gas3) and disruption of β1 integrin function. Mol. Cancer Res..

[22] Mittendorf E.A., Philips A.V., Meric-Bernstam F., Qiao N., Wu Y., Harrington S., Su X., Wang Y., Gonzalez-Angulo A.M., Akcakanat A. (2014). PD-L1 expression in triple-negative breast cancer. Cancer Immunol. Res..

[23] Antony G.R., Augustine P., Parambil S.T., Littleflower A.B., Kattoor J., Krishna K.M.J., Subhadradevi L. (2023). Immunohistochemical expression of PD-L1 and MDR1 in breast tumors: Association with clinico-pathological parameters and treatment outcome. Clin. Exp. Med..

[24] Chatterjee K., Zhang J., Honbo N., Karliner J.S. (2010). Doxorubicin cardiomyopathy. Cardiology.

[25] Costa R.L.B., Gradishar W.J. (2017). Triple-Negative Breast Cancer: Current Practice and Future Directions. J. Oncol. Pract..

[26] Zagami P., Carey L.A. (2022). Triple negative breast cancer: Pitfalls and progress. NPJ Breast Cancer.

[27] Bianchini G., Balko J.M., Mayer I.A., Sanders M.E., Gianni L. (2016). Triple-negative breast cancer: Challenges and opportunities of a heterogeneous disease. Nat. Rev. Clin. Oncol..

[28] So J.Y., Ohm J., Lipkowitz S., Yang L. (2022). Triple negative breast cancer (TNBC): Non-genetic tumor heterogeneity and immune microenvironment: Emerging treatment options. Pharmacol. Ther..

[29] Jin C., Li W., Liu B., Cao L.-Q., Stefan S.M., Yuan L., Yu X., Shi L., Yu H. (2026). Emerging trends and converging evidence in tumor evolution: A comprehensive review. Drug Resist. Updat..

[30] Kotsifaki A., Kalouda G., Karalexis E., Stathaki M., Metaxas G., Armakolas A. (2025). Emerging Breast Cancer Subpopulations: Functional Heterogeneity Beyond the Classical Subtypes. Int. J. Mol. Sci..

[31] Nalla L.V., Kanukolanu A., Yeduvaka M., Gajula S.N.R. (2025). Advancements in Single-Cell Proteomics and Mass Spectrometry-Based Techniques for Unmasking Cellular Diversity in Triple Negative Breast Cancer. Proteom. Clin. Appl..

[32] Maggisano V., D’amico M., Aquila S., Giordano F., Battaglia A.M., Chimento A., Biamonte F., Russo D., Pezzi V., Bulotta S. (2025). IL-20 Subfamily Biological Effects: Mechanistic Insights and Therapeutic Perspectives in Cancer. Int. J. Mol. Sci..

[33] Emdad L., Bhoopathi P., Talukdar S., Pradhan A.K., Sarkar D., Wang X.-Y., Das S.K., Fisher P.B. (2020). Recent insights into apoptosis and toxic autophagy: The roles of MDA-7/IL-24, a multidimensional anti-cancer therapeutic. Semin. Cancer Biol..

[34] Emdad L., Lebedeva I.V., Su Z.-Z., Sarkar D., Dent P., Curiel D.T., Fisher P.B. (2007). Melanoma differentiation associated gene-7/interleukin-24 reverses multidrug resistance in human colorectal cancer cells. Mol. Cancer Ther..

